# Intestinal Microbiome-Metabolome Responses to Essential Oils in Piglets

**DOI:** 10.3389/fmicb.2018.01988

**Published:** 2018-08-28

**Authors:** Yuan Li, Xiongfeng Fu, Xin Ma, Shijie Geng, Xuemei Jiang, Qichun Huang, Caihong Hu, Xinyan Han

**Affiliations:** ^1^Key Laboratory of Animal Nutrition and Feed Science in East China, Ministry of Agriculture, College of Animal Sciences, Zhejiang University, Hangzhou, China; ^2^Department of Life Science, Longyan University, Longyan, China

**Keywords:** essential oils, colon, microbiota, metabolic profiles, weaned piglets

## Abstract

This study investigated the effects of dietary essential oils (EOs) on intestinal microbial composition and metabolic profiles in weaned piglets. The piglets were fed the same basal diet supplemented with EOs (EO) or without EOs (Con) in the current study. The results showed that the body weight gain was significantly increased, while the diarrhea incidence was significantly reduced in the EO group. In addition, EOs could modify the intestinal microbial composition of weaned piglets. The relative abundances of some beneficial bacterial species such as *Bacilli*, *Lactobacillales*, *Streptococcaceae*, and *Veillonellaceae* were significantly increased in the EO group. Metabolomics analysis indicated that protein biosynthesis, amino acid metabolism, and lipid metabolism were enriched in the EO group. And correlation analysis demonstrated that some gut bacterial genera were highly correlated with altered gut microbiota-related metabolites. Taken together, this study indicated that dietary EOs not only altered microbial composition and function but modulated the microbial metabolic profiles in the colon, which might help us understand EOs’ beneficial effects on intestinal health of weaned piglets.

## Introduction

It is known that vast and diverse microbiota at an amount of approximately 100 trillion inhabits the gastrointestinal tract in human being and animal. The intestinal microbiota plays a vital role in host health, including nutrient absorption and metabolism, the host immune defense system development, the intestinal epithelium differentiation, and intestinal mucosal barrier maintenance (Garrett et al., 2010). Accumulating evidences demonstrate that intestinal microbiota participates in many metabolic pathways, such as the nutrient digestion and absorption, lipid metabolism and amino acid synthesis (Li et al., 2008). The intestinal microbiota and their metabolites are important environmental factors, which affect the immune system, obesity, cardiovascular disease, and brain activity of the host (Lee and Hase,  2014; Postler and Ghosh,  2017).

The host gut–microbial relationship is dynamic and highly susceptible to numerous environmental factors, especially diet. Essential oils (EOs), a class of volatile aromatic molecules, are one of the main kinds of natural plant extracts. They are extracted from plant’s flowers, leaves, stems, roots, seeds, or fruits by steam distillation, extrusion, or solvent extraction (Brenes and Roura,  2010). However, they are not simple compounds, rather a mixture of various compounds (mainly terpenes and terpene derivatives) (Başer and Demirci,  2007). In terms of biological activity and effects, each individual chemical constituent has its own characteristic properties. They are used widely in cosmetics, medicine, and food industry (Ran et al., 2016). In animal science, EOs have attracted more attention as an alternative for antibiotics used in feed (Zeng et al., 2015) due to the safety and little residual (Ran et al., 2016). Studies showed that EOs could increase the weight gain and improve resistance to infection in swine and poultry (Zeng et al., 2015). Besides, EOs showed beneficial effects on antioxidant status, intestinal morphology, and barrier in animals (Giannenas et al., 2014). Previous studies found that carvacrol and thymol could reduce the amount of *E. coli* and *Clostridium perfringens*, increase the number of *Lactobacilli* in the intestine of broilers (Di Pasqua et al., 2007; Cho et al., 2014). However, the effects of EOs containing carvacrol and thymol mixture on the intestinal microbiota and microbial metabolites in the intestine of pigs are limited.

In the present study, weaned piglets were selected as experimental animal. We hypothesized that dietary EOs could shape intestinal microbiota and its metabolites in piglets. An integrated approach combination of 16S rRNA gene sequencing and the mass spectrometry (MS)-based metabolomic technique was used to investigate the effects of dietary EOs on microbial composition and metabolites in the colon of weaned piglets.

## Materials and Methods

### Animals and Experimental Design

The present study followed the institutional and national guidelines for the care and use of animals. All experimental procedures involving animal care and sampling were approved by the Ethics Committee for Animal Experimentation of Zhejiang University under permit number SYXK (Zhejiang) 2012-0178. A total of 96 barrows (Duroc × Landrace × Yorkshire) weaned at 26 ± 1 d of age, with an average initial body weight of 7.29 ± 0.25 kg, were randomly allocated to two treatments. Each treatment was replicated three times, with 16 piglets per replicate (i.e., pen). All piglets fed the same basal diet supplemented EO or Con (62.5 mg/kg carvacrol of feed and 7.5 mg/kg thymol of feed, EO). EOs used in this study were provided by Zhong Nong Mu BIO-Pharmaceutical Co., Ltd. (Fujian, China). The lot number was 20150527. The basal diet was formulated to meet or exceed the nutrient requirements recommended by NRC (2012, **Table 1**). Pigs were housed in standard pens equipped with a fully slatted mesh floor and duckbill drinking fountains. All pigs were given *ad libitum* access to feed and water during the feeding trial. The trial lasted 30 days.

**Table 1:** Ingredient composition and nutritional levels of basic diet.

Item		Nutritional level^2^	
Corn	567	Digestible Energy (MJ/kg)	13.98
Puffed soybean	130	Crude protein (g/Kg)	191
Soybean meal	155	Crude fat (g/Kg)	65.7
Sucrose	10	Ash (g/Kg)	53.1
Fish meal	30	Moisture (g/Kg)	110.3
Whey powder	30	Calcium (g/Kg)	9.3
Plasma protein powder	10	Phosphorus (g/Kg)	6.5
Soybean oil	10	Lysine (g/Kg)	11.5
Stone powder	8	Methionine (g/Kg)	3.0
Calcium dihydrogen phosphate	10		
Vitamin premix^1^	40		


*^*1*^Provided per kilogram of diet: 16,000IU vitamin A, 4000IU vitamin D_*3*_, 100IU vitamin E, 0.5 mg vitamin K_*3*_, 2 mg vitamin B_*1*_, 4.5 mg vitamin B_*2*_, 7 mg vitamin B_*6*_, 0.03 mg vitamin B_*12*_, 0.2 mg biotin, 10 mg folic acid, 30 mg nicotinic acid, 22 mg pantothenic acid; 85 mg Fe(FeSO_*4*_), 100 mg Cu(CuSO_*4*_), 0.3 mg Mn(MnSO_*4*_), and 0.14 mg I(CaI_*2*_). ^*2*^The data regarding crude protein, crude fat, crude ash, moisture, calcium, and total phosphorus are measured values, the others are calculated values.*

Piglets were weighed at the beginning and the end of the experiment. And the feed consumption was record daily. Average daily gain (ADG), average daily feed intake, and feed:gain ratio were calculated. All piglets were checked daily for their health status by fecal consistency. The fecal consistency was scored as follows: 1 = normal, 2 = pasty, 3 = watery, and 4 = watery with color changes (Maenner et al., 2011). The incidence of diarrhea was defined when the feces were watery and watery with color changes (Li et al., 2012). Diarrhea incidence (%) = sum (diarrhea piglet × number of days on diarrhea)/(number of piglets in the pen × number of days of trials) × 100%.

### Sample Collection

At the end of the feeding trial, 12 pigs (without feed for 12 h, two piglets per pen and six piglets each group) were randomly selected and euthanized with sodium pentobarbital (50 mg/kg BW). The samples of colonic content (from the middle of the colon) were collected. These samples were placed into 1.5 mL sterile polypropylene tubes, then snap frozen immediately in liquid nitrogen and stored at -80°C until further microbiome and metabolome analysis.

### DNA Extraction and 16S rRNA Gene Amplicon Sequencing

Total bacteria DNA was extracted from the samples of colonic contents by using Bacterial Genomic DNA Extraction Kit (DP302) [TIANGEN Biotech (Beijing) Co., Ltd., China] according to manufacturer’s instruction, and was stored at -80°C before further analysis. Sequencing was performed at the Annoroad Gene Technology. Briefly, DNA was amplified by using the 341F-805R primer set (341F: 5′-CCTACGGGNGGCWGCAG-3′, 805R: 5′-GACTACHVGGGTATCTAATCC-3′), which targeted the (V3 + V4) region of the bacterial 16S rDNA. PCR reaction was performed using Phusion High-Fidelity PCR Master Mix [New England Biolabs (Beijing) Ltd., China] with the following condition: 95°C for 3 min (1 cycle), 95°C for 30 s/55°C for 30 s/72°C for 30 s (35 cycles), and a last step of 16°C for 2 min [16]. Then, mixture PCR product was purified with GeneJET Gel Extraction Kit (Thermo Fisher Scientific). Sequencing libraries were generated using NEB Next Ultra DNA Library Prep Kit for Illumina (NEB, United States) following manufacturer’s recommendations and index codes were added. The library quality was assessed on the Qubit @ 2.0 Fluorometer (Life Technologies, CA, United States) and Agilent Bioanalyzer 2100 system. At last, the library was sequenced on an Illumina MiSeq platform and 250 bp paired-end reads were generated.

### Sequence Analysis

For the sequences obtained by the sequencing, the data filtering were completed by removing low quality base, Ns, joint contaminating sequences, and other processes, and obtained a trusted target sequence for subsequent analysis. Filtered sequences were called Clean Reads. First, the corresponding Read1 and Read2 (the sequence fragments of Read1 and Read2 were obtained in the two directions from the 5′ and 3′ ends, respectively) of the paired-end sequencing were concatenated by the sequence splicing method PEAR (Zhang et al., 2013); then, the sequence after splicing was analyzed using software QIIME version 1.8.0 (Caporaso et al., 2010; Vasileiadis et al., 2012; Ahn et al., 2013), including the extraction of operational taxonomic units (OTUs), overlapping analysis of OTUs, clustering analysis, Alpha diversity analysis, Beta diversity analysis, and so forth (Adler et al., 2013).

Operational taxonomic units were clustered with a 97% similarity threshold. Alpha diversity analysis included Shannon diversity index, Chao1 richness estimate, and observed species richness. Principal component analysis (PCA) was performed at the genus level (Han et al., 2016), and the results were visualized using the Statistical Analysis of Metagenomic Profiles (STAMP) version 2.1.3 program (Parks et al., 2014). Linear discriminant analysis coupled with effect size (LEfSe) was performed to identify the bacterial taxa differentially represented between groups at genus or higher taxonomy levels (Segata et al., 2011). Based on the precalculated Greengenes (v 13.5) database, PICRUSt was performed on the abundance predictions of the Kyoto Encyclopedia of Genes and Genomes (KEGG) orthologs and KEGG pathways of bacterial communities (Langille et al., 2013). The functional differences between two groups were compared through the software STAMP (Parks et al., 2014). Two-sided Welch’s *t* -test and Benjamini–Hochberg FDR correction were used in two-group functional prediction analysis. In addition, the prediction accuracy of PICRUSt was evaluated by the Nearest Sequenced Taxon Index (NSTI), with lower value indicating a higher accuracy of prediction (Hu et al., 2016).

### Sample Preparation for Gas Chromatograph-Mass Spectrometry (GC-MS) Analysis

The samples of colonic content were prepared using the following procedure. First, 0.4 mL extraction liquid (*V methanol:V chloroform* = 3:1) and 20 μL of L-2-Chlorophenylalanine (1 mg/mL stock in dH_2_O) were added to a 100 mg sample, homogenized in ball mill for 4 min at 40 Hz, then ultrasound treated for 5 min (incubated in ice water); centrifuged at 4°C, 12 000 rpm for 10 min; 0.35 mL of supernatant was then transferred into a fresh 2 mL GC/MS glass vial. After the extracts were dried using a vacuum concentrator without heating, 40 μL of Methoxyamine hydrochloride (20 mg/mL in pyridine) was added and mixed gently; the solution was then incubated at 80°C for 30 min. Subsequently, 60 μL of the BSTFA regent (1% TMCS, v/v) was added to each sample, followed by incubation at 70°C for 2 h; the samples were then subjected to detection by GC-TOF/MS.

### GC-MS Analysis of Metabolite Profiles

GC-TOF/MS analysis was performed using an Agilent 7890 gas chromatograph system coupled with a Pegasus HT time-of-flight mass spectrometer. The system utilized a DB-5MS capillary column coated with 5% diphenyl cross-linked with 95% dimethylpolysiloxane (30 m × 250 μm inner diameter, 0.25 μm film thickness; J&W Scientific, Folsom, CA, United States). A 1 μL aliquot of the analyte was injected in splitless mode. Helium was used as the carrier gas, the front inlet purge flow was 3 mL min^-1^, and the gas flow rate through the column was 1 mL min^-1^. The initial temperature was kept at 50°C for 1 min, then raised to 290°C at a rate of 10°C min^-1^, then kept for 15 min at 290°C. The injection, transfer line, and ion source temperatures were 280, 280, and 220°C, respectively. The energy was -70 eV in electron impact mode. The MS data were acquired in full-scan mode with the m/z range of 50–500 at a rate of 20 spectra per second after a solvent delay of 366 s (Garcia and Barbas,  2011).

### GC-MS Data Acquisition and Processing

Chroma TOF 4.3X software of LECO Corporation and LECO-Fiehn Rtx5 database were used for raw peaks exacting, the data baselines filtering and calibration of the baseline, peak alignment, deconvolution analysis, peak identification, and integration of the peak area (Kind et al., 2009). The retention time index (RI) method was used in the peak identification, and the RI tolerance was 5,000. First of all, 704 peaks were detected and 283 metabolites could be left through interquartile range denoising method, and then, missing values of raw data were filled up by half of the minimum value. In addition, sum of area normalization method was employed in this data analysis. The resulted three-dimensional data involving the peak number, sample name, and normalized peak area were fed to SIMCA14.1 software package (Umetrics, Umea, Sweden) for three dimensional-principal component analysis (3D-PCA) and orthogonal projections to latent structures-discriminate analysis (OPLS-DA). 3D-PCA showed the distribution of origin data. In order to obtain a higher level of group separation and get a better understanding of variables responsible for classification, OPLS-DA was applied. Afterward, the parameters for the classification from the software were *R*^2^*Y* = 0.984 and *Q*^2^ = 0.614, which were stable and good to fitness and prediction. To refine this analysis, the first principal component of variable importance projection (VIP) was obtained. The VIP values exceeding 1.0 were first selected as changed metabolites (Sun et al., 2015). The remaining variables were then assessed by Student’s *t* -test. If *P* > 0.05, variables were discarded between two comparison groups. In addition, commercial databases, including KEGG^1^ and NIST^2^ was utilized to search for the pathways of metabolites. A free and web-based tool, MetaboAnalyst, which use the high-quality KEGG metabolic pathway as the backend knowledgebase, was for pathway analysis^3^ (Xia et al., 2009).

### Statistical Analyses

All data except function prediction were analyzed by SPSS 20.0 using Student’s *t* -test, and the results of growth performance and diarrhea incidence were presented as means ± SEM. A value of *P* < 0.05 was considered statistically significant. The correlation matrix between gut bacterial species and microbial metabolites was generated using Spearman’s correlations coefficient (Lu et al., 2014). Hierarchical clustering heat map, metabolic pathway enrichment and correlation matrix were visualized by using R. All statistical analysis in the study was carried out by specialized statisticians.

## Results

### Weight Gain and Diarrhea Incidence of Weaned Piglets

Weight gain and diarrhea incidence of two groups were presented in **Table 2**. The results showed that compared with the Con group, ADG was significantly increased (*P* < 0.05), while feed:gain and diarrhea incidence was significantly decreased in the EO group (*P* < 0.05).

**Table 2:** Weight gain and diarrhea incidence of weaned piglets fed with or without dietary EO^1^.

Variable	Con	EO	SEM	*P* -value
Initial body weight, kg	7.34 ± 0.13	7.23 ± 0.16	0.21	0.61
Final body weight, kg	20.5 ± 0.48^b^	22.6 ± 0.43^a^	0.65	0.03
ADG^1^, g/d	356 ± 9.70^b^	416 ± 7.36^a^	12.2	<0.01
Average daily feed intake, g/d	816 ± 38.8	870 ± 27.3	47.4	0.32
Feed:gain	2.29 ± 0.05^a^	2.09 ± 0.04^b^	0.07	0.04
Diarrhea, %^2^	11.2 ± 1.60^a^	5.00 ± 0.21^b^	0.95	0.03
Survival rate, %^3^	95.8 ± 4.17	100 ± 0.00	4.17	0.42


*^*1*^Values were Means ± SEM. ^*ab*^Means in the same row with different superscript letters were significantly different (*P* < 0.05). ^*1*^ADG was calculated as weight gain (final body weight-initial body weight) divided by the number of treatment days. ^*2*^Diarrhea index (%) = sum (piglet with diarrhea × number of days on diarrhea)/(animal number in the pen × number of days of trials) × 100. ^*3*^Survival rate (%) = number of surviving piglets/animal number in the pen × 100. Con: the basal diet; EO: the basal diet supplemented with essential oils.*

### The Diversity and Composition of Gut Microbiota

**FIGURE 1:**
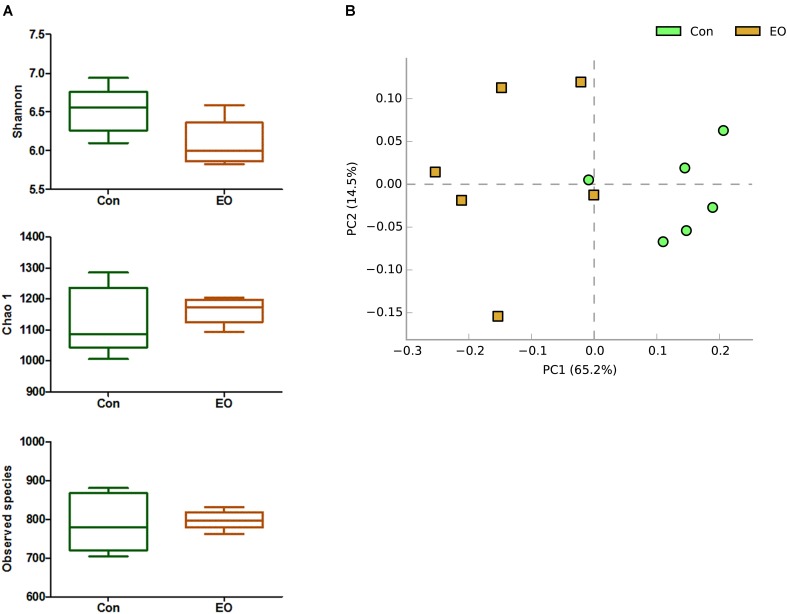
Differences in bacterial community diversity, richness, and structures in the colon of weaned piglets fed with or without dietary EO. **(A)** Community diversity and richness between Con and EO group. **(B)** Principal components analysis (PCA) of bacterial community structure between Con and EO group. Each symbol represented each gut microbiota. Green symbols represented Con group and brown symbols represented EO group. Con: the basal diet; EO: the basal diet supplemented with essential oils.

**FIGURE 2:**
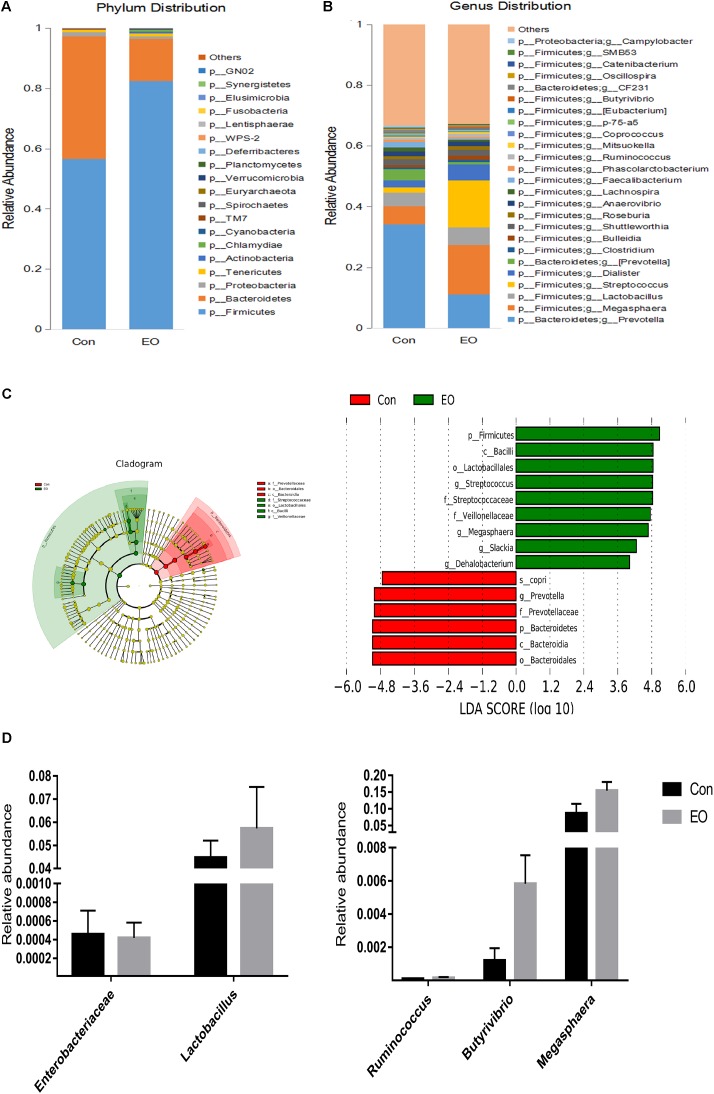
Changes of microbial composition in the colon of weaned piglets fed with or without dietary EO. Microbial composition at the phylum level **(A)** and genus level **(B)** each bar represented the average relative abundance of each bacterial taxon within a group. **(C)** Cladogram and LDA value distribution histogram. Bacterial taxa significantly differentiated between Con and EO group identified by linear discriminant analysis coupled with effect size (LEfSe) using the default parameters. **(D)** Difference of the relative abundances of *Lactobacillus*, *Enterobacteriaceae*, *Ruminococcus*, *Butyrivibrio*, and *Megasphaera* between Con and EO group. Con: the basal diet; EO: the basal diet supplemented with essential oils.

An average of 38,146 high-quality sequences per sample was obtained from 12 colonic content samples. Further, an average of 840 OTUs based on a 97% sequence similarity was identified from these sequences in colonic contents. The alpha diversity was estimated through Shannon diversity index, Chao1 richness estimate, and observed species richness. **Figure 1A** showed that there were no differences in diversity indices (Shannon) and richness (Chao1 and Observed species) between two groups (*P* > 0.05). As shown in **Figure 1B**, the Con and EO group were well separated, with 65.2 and 14.5% variation explained by principal component PC1 and PC2, respectively. The results suggested that the composition of gut microbiota in weaned piglets was changed by EO supplement. **Figures 2A,B** showed the microbial composition of weaned piglets in two groups. **Figure 2A** showed that *Firmicutes* and *Bacteroidetes* were predominant phyla in colonic microbiota, followed by Proteobacteria, Tenericutes, and Actinobacteria. Relative abundance of gut microbiota composition at the genus level was shown in **Figure 2B**. The result showed that *Prevotella*, *Lactobacillus*, Dialister, and *Clostridium* were the predominant genera.

A cladogram representative of the structure of the colonic microbiota and the predominant bacteria was shown in **Figure 2C**; the greatest differences in taxa between the two groups were displayed. EO supplementation significantly promoted the relative abundance of *Firmicutes*, *Bacilli*, *Lactobacillales*, *Streptococcaceae*, *Veillonellaceae*, *Megasphaera*, *Slackia*, and *Dehalobacterium*, while it reduced the relative abundance of *Bacteroidales*, *Bacteroidia*, *Bacteroidetes*, *Prevotellaceae*, and *Prevotella* (**Figure 2C**). **Figure 2D** showed that compared with the Con group, the relative abundance of *Enterobacteriaceae* was decreased, the relative abundance of *Lactobacillus*, *Ruminococcus*, *Butyrivibrio*, and *Megasphaera* were increased in the EO group, although the differences were not significant (*P* > 0.05).

### Predicted Function of Intestinal Microbiota

**FIGURE 3:**
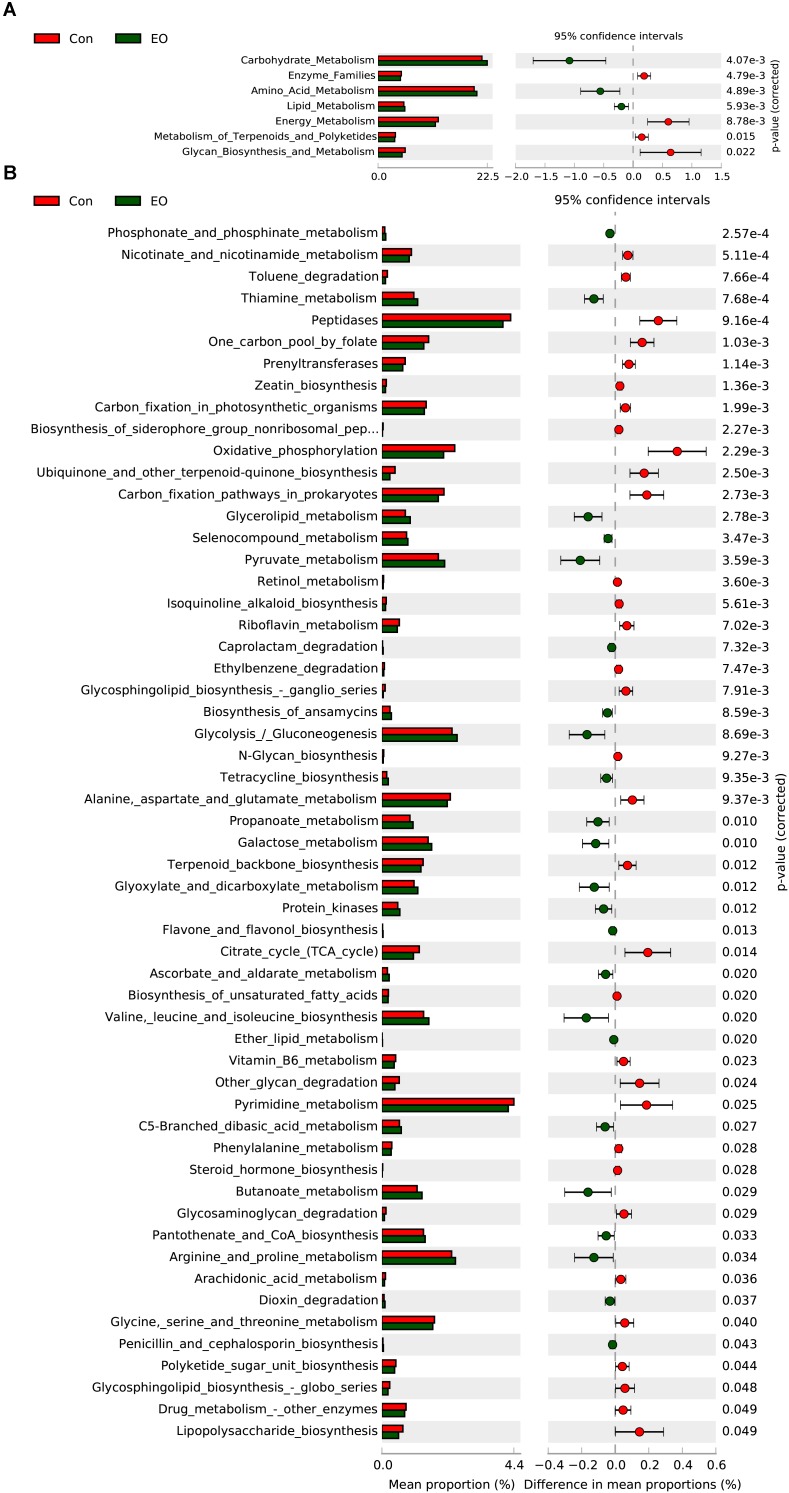
Microbial function prediction in the colon of weaned piglets fed with or without dietary EO. The second level **(A)** and third level **(B)** of KEGG pathway were showed in the extended error bar. The *P* -values were shown at right. Con: the basal diet; EO: the basal diet supplemented with essential oils.

In order to evaluate the functional capacity of the intestinal bacterial community between the Con group and the EO group, PICRUSt method was performed to analyze the KEGG pathways compositions of intestinal microbiota. As shown in **Figure 3A**, the second level KEGG pathways analysis showed that carbohydrate metabolism, amino acid metabolism, lipid metabolism was enriched, while enzyme family, energy metabolism were decreased in the EO group. Consistent with the results of the second level KEGG pathways, **Figure 3B** showed that the relative abundances of genes involved peptidases, glycolysis/gluconeogenesis, pyruvate metabolism, propanoate metabolism, galactose metabolism, butanoate metabolism, valine, leucine, and isoleucine degradation, arginine and proline metabolism were significantly increased by dietary EO (*P* < 0.05). While, the abundance of genes involved in citrate cycle, pyrimidine metabolism, and drug metabolism other enzymes were decreased (*P* < 0.05).

### Intestinal Metabolites and Metabolic Pathways

**FIGURE 4:**
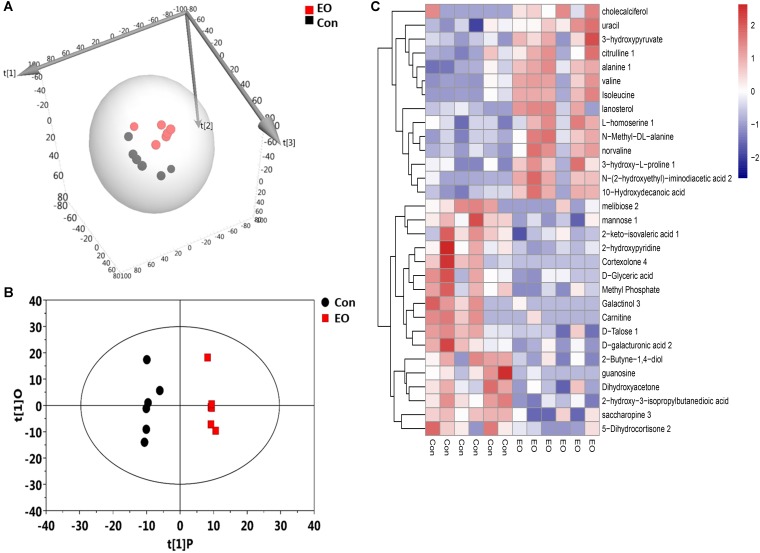
3D-PCA score map, OPLS-DA score plots, and hierarchical clustering heat map derived from the GC-TOF/MS metabolic profiles in the colon of weaned piglets fed with or without dietary EO. **(A)** 3D-PCA score map showed the distribution of origin data. **(B)** OPLS-DA score plots showed significantly separated clusters between EO and Con group. Black represented Con group and red represented EO group. **(C)** Hierarchical clustering heat map constructed using molecular features with 1-fold changes (*P* < 0.05) showed a consistent clustering pattern within individual groups. Con: the basal diet; EO: the basal diet supplemented with essential oils.

To further explore the influence of EO supplementation on intestinal microbiota, we analyzed the metabolites of the colon contents in the two groups. A total of 704 untargeted peaks were detected by GC-MS and 283 metabolites were annotated. These metabolites, including amino acids, carbohydrates, organic acids, lipids, nucleotides, and others, were involved in multiple biochemical processes in the colon of weaned piglets. 3D-PCA score plot derived from the GC-TOF/MS metabolic profiles of colonic contents showed separation between EO and Con group (**Figure 4A**). **Figure 4B** showed a clear separation and discrimination, which indicated that the OPLS-DA model could be used to identify the difference between the two groups. The hierarchical clustering heat map in **Figure 4C** also showed similar clustering patterns of detected molecular features within each group.

**FIGURE 5:**
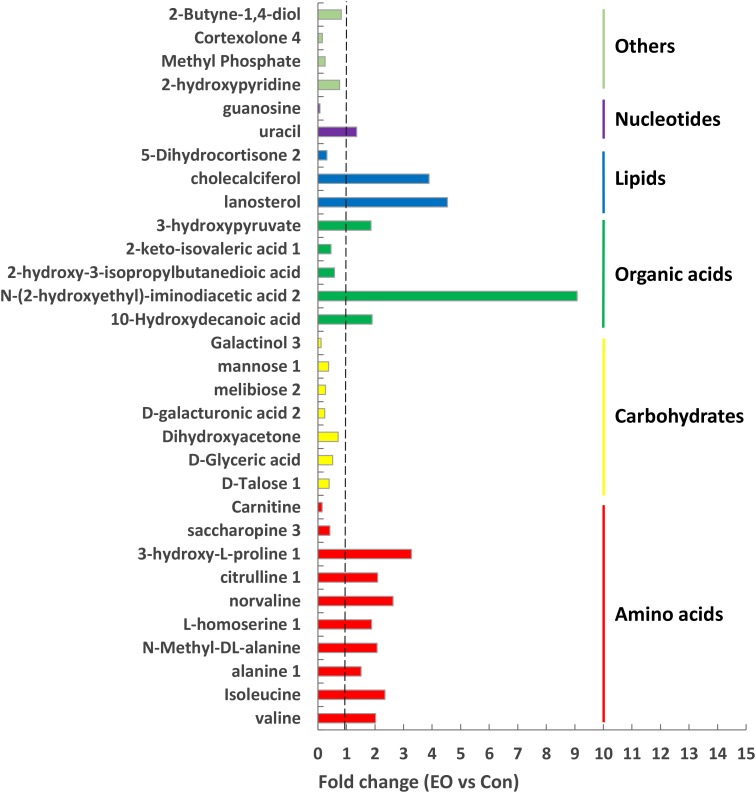
Significantly differential metabolites in the colon of weaned piglets fed with or without dietary EO. Metabolites accountable for class discrimination with VIP > 1 and *P* < 0.05 were listed. Con: the basal diet; EO: the basal diet supplemented with essential oils.

**FIGURE 6:**
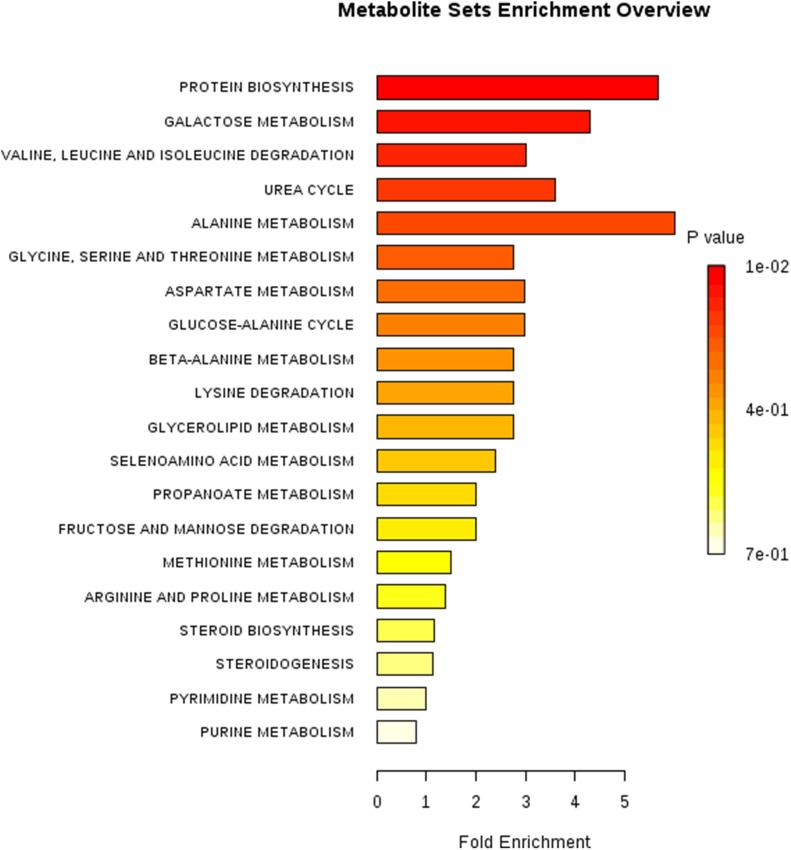
Metabolic pathway enrichment analysis. Overview of metabolites that were enriched in the colon of weaned piglets fed with or without dietary EO. Con: the basal diet; EO: the basal diet supplemented with essential oils.

To identify differential compounds between two groups, the parameters of VIP > 1 and *P* < 0.05 were used as a criteria. In the colon, 14 compounds [uracil, valine, isoleucine, alanine 1, N-Methyl -DL-alanine, L-homoserine 1, norvaline, citrulline 1, lanosterol, 3-hydroxy-L-proline 1, N-(2-hydroxyethyl)-iminodiacetic acid 2, cholecalciferol, 10-Hydroxydecanoic acid, 3-hydroxypyruvate] were increased, while 17 compounds (D-Talose 1, 2-hydroxypyridine, D-Glyceric acid, Dihydroxyacetone, Methyl Phosphate, D-galacturonic acid 2, guanosine, melibiose 2, mannose 1, Cortexolone 4, 2-Butyne-1,4-diol, saccharopine 3, Galactinol 3, 2-hydroxy-3-isopropylbutanedioic acid, 5-Dihydrocortisone 2, Carnitine, and 2-keto-isovaleric acid 1) were decreased in the EO group compared with the Con group (**Figure 5**). Further metabolic pathway enrichment analysis showed that EO treatment played an important role in protein biosynthesis, galactose metabolism, amino acid metabolism, urea cycle, and lipid metabolism (**Figure 6**).

### Correlation Between Microbiota Community and Metabolites

**FIGURE 7:**
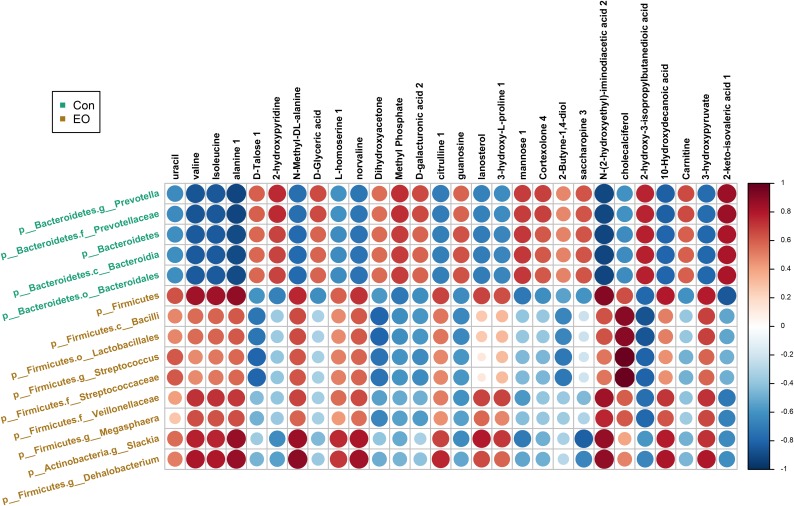
Correlation between microbiota and metabolites in the colon of weaned piglets fed with or without dietary EO. The color was according to the Spearman correlation coefficient distribution. Red represented significant positive correlation (*P* < 0.05), blue represented significantly negative correlation (*P* < 0.05), and white represented that the correlation was not significant (*P* > 0.05). Con: the basal diet; EO: the basal diet supplemented with essential oils.

Metabolites with VIP > 1 and bacteria with significant differences between Con and EO group were used for the Spearman’s correlation analysis. As shown in **Figure 7**, the relative abundance of *p_Firmicutes*, *p_Firmicutes.c_Bacilli*, *p_Firmicutes.o_Lactobacillales*, *p_Firmicutes. g_Streptococcus*, and *p_Firmicutes*. *f_Veillonellaceae*, was positively correlated with valine, Isoleucine, alanine 1, N-Methyl -DL-alanine, norvaline, N-(2-hydroxyethyl)-iminodiacetic acid, and 3-hydroxypyruvate (*P* < 0.05), while negatively correlated with dihydroxyacetone, guanosine, cholecalciferol, and 2-hydroxy-3-isopropylbutanedioic acid (*P* < 0.05). The relative abundance of *p_Bacteroidetes*, *p_Bacteroidetes*, and *g_Prevotella* showed negative correlations with valine, isoleucine, alanine 1, N-Methyl -DL- alanine, norvaline, N-(2-hydroxyethyl)-iminodiacetic acid 2, 10-Hydroxydecanoic acid, and 3-hydroxypyruvate (*P* < 0.05), and positive correlations with dihydroxyacetone, guanosine, cholecalciferol, and 2-hydroxy-3-isopropyl butanedioic acid in the Con group (*P* < 0.05).

## Discussion

Weaning piglets are usually suffered to psychological, physiological, and social stress, which would lead to transient anorexia, post-weaning diarrhea and consequent growth retardation (Pié et al., 2004; Lallès et al., 2007). Previous studies showed that dietary thymol-containing plant extracts reduced feed:gain ratio, increased feed intake and weight gain in weaned piglets (Li et al., 2012). The present study showed a similar result. In addition, decreased diarrhea incidence was observed in EOs treated piglets. The improvement in feed intake is mainly due to the pleasant odor and flavor of EOs (Cao et al., 2010). While nutrient digestion was not measured in the current study, Windisch et al. (2008) found that EOs might increase the activity of digestive enzymes and nutrient absorption, thus promoting the weight gain (Windisch et al., 2008). And thymol exerts antimicrobial and anti-inflammatory effects, which may be associated with a decrease in the rate of diarrhea (Lee et al., 2004). These results demonstrated that EOs could promote the growth performance of weaned piglets.

Gut contains a huge number of microorganisms, which play an important role in human and animal health. EOs had been appreciated for their beneficial effects on antibacterial activity and the main active ingredient of antibacterial was phenolic substances. Study showed that carvacrol and thymol had a synergistic effect in terms of antimicrobial (Brenes and Roura,  2010). In this study, we found that EOs modified the intestinal microbiota. The results indicated that there were no differences in diversity and richness indexes of the colonic microbiota between two groups. However, previous study found that the diversity indexes of gut microbiota in EO-supplemented piglets was not impacted, but the richness indexes including Chao1 and observed OTUs were increased (Ran et al., 2016). The differences of the two studies might be due to the different animal models used in the work. Consistent with previous research (Hu et al., 2016), *Firmicutes* and *Bacteroidetes* were predominant in gut microbiota. However, we found that the relative abundance of *Firmicutes* significantly was increased and the relative abundance of *Bacteroidetes* was decreased after EOs supplement in this study. These results suggested that EO treatment significantly changed the structure of the gut microbiota in weaned piglets. Study showed that the energy absorption was enhanced in germ-free mice when the number of *Firmicutes* was larger than that of *Bacteroidetes* (Turnbaugh et al., 2006). Similarly, increased *Firmicute* abundance might enhance the energy absorption of piglets fed with EOs in this study. *Lactobacillales* within *Bacilli* are recognized beneficial bacteria, which helps maintain normal intestinal function and promote the health of body (Zalán et al., 2011). *Streptococcaceae* and *Streptococcus* were found to be negatively associated with inflammation in previous studies (Ferná ndez et al., 2016). The higher relative abundance of *Streptococcaceae* in the EO group indicated that intestinal health of weaned piglets was improved in this study. *Veillonellaceae* comprises several acetate and propionate producers and was increased in the EO group. In addition, *Megasphaera*, *Butyrivibrio*, and *Ruminococcus* had an increased trend in EO group in this study. These bacteria genera are short-chain fatty acids (SCFAs)-producing bacteria, which were confirmed to play a key role in colonic health (Louis and Flint,  2009; Bonder et al., 2016). Ouwehand et al. (2010) found that carvacrol and thymol could inhibit the colonization of *E. coli* and increase the ratio of *Lactobacillus* and *Enterobacteriaceae*. Similarly, we found an increased trend on *Lactobacillus* and a decreased trend on *Enterobacteriaceae* in the EO group in the present study. These results suggested that EOs modified the intestinal microbiota in weaned piglets by promoting the growth of some beneficial bacteria.

Intestinal microbiota plays a key role in energy metabolism of the host. Non-digestible carbohydrates are degraded by colonic microbiota to produce metabolic end products (Sun et al., 2016). Intestinal microbiota interacts with numerous physiological functions and the pathogenesis of various diseases in the host through its metabolic products (Malmuthuge and Guan,  2016). Besides affecting the composition of intestinal microbiota, EOs also altered the metabolism of intestine microbiota. In this study, microbiota function prediction showed that the colonic microbiota in the EO group had higher enrichments of functions involved in carbohydrate metabolism, amino acid metabolism, and lipid metabolism. Specially, enriched propanoate metabolism and butanoate metabolism of intestinal microbiota were found in the EO group. These metabolic pathways produced some SCFAs such as propionate and butyrate, which were known to protect the host against colonic diseases, improve the gut barrier function and exhibit anti-inflammatory effects (Fukuda et al., 2011). Metabolic pathway enrichment analysis indicated that protein biosynthesis, galactose metabolism, amino acid metabolism, and lipid metabolism played an important role in the EO group. Based on PICRUSt and GC-MS analysis, we found that EOs mainly altered the amino acid metabolism. Changed metabolites also confirm this result. The increased concentrations of several amino acids in the EO group, such as valine, isoleucine, alanine, and L -homoserine, suggested higher nitrogen sources existed for fermentation. The possible reason is that the increase in carbohydrate metabolism results in lower carbon source and higher nitrogen of substrates for microbes in the colon.

The abundance of intestinal microbiota was believed to be correlated with energy harvesting and weight gain (Yan et al., 2017). Previous study showed that there was a possible link between the intestinal microbiota and feed efficiency in pigs (McCormack et al., 2017). As shown by Spearman’s correlation analysis in this study, changes in intestinal microbial abundance induced by EOs resulted in a shifted metabolome of intestinal microbiota. *Firmicutes* genera like *Streptococcus* and *Megasphaera* were positively correlated with valine, isoleucine, alanine 1, N-methyl -DL-alanine, and norvaline in colon of weaned piglets fed dietary EOs. *Firmicute* was proved to be associated with energy absorption (Turnbaugh et al., 2006). Amino acids are not only an important raw material for proteins synthesis, but provide a material basis for promoting growth and normal metabolism. The positive correlation between *Firmicutes* genera and some amino acids suggested that higher production of microbiota-derived amino acids may be positively correlated with the higher performance of piglets fed with EO.

## Conclusion

In conclusion, the current study showed intestinal microbiome–metabolome responses to EOs supplementation in weaned piglets. The results demonstrated that EOs shaped the intestinal microbiota in weaned piglets through promoting the growth of some benefit bacterial species such as *Bacilli*, *Lactobacillales*, *Streptococcaceae*, *Veillonellaceae*, and *Megasphaera* in the colon. Moreover, multiple metabolic pathways especially amino acid metabolism, carbohydrate metabolism, and lipid metabolism of intestinal microbiota were substantially altered by EO supplementation. And correlation analysis identified that some gut bacterial genera were highly correlated with altered gut microbiota–related metabolites. Taken together, these data indicated that EO supplementation not only modified the microbial composition and function but influenced the microbial metabolic profiles. These findings might provide insights into future application of the alternative strategy for improving the health in weaned piglets.

## Author Contributions

XH and CH designed the experiments. XF performed the experiments and edited the manuscript. YL and SG performed the experiments. XM and XJ analyzed the data. YL and XF wrote the manuscript, which was edited by SG, CH, XM, XJ, and XH. All authors read and approved the final manuscript.

## Conflict of Interest Statement

The authors declare that the research was conducted in the absence of any commercial or financial relationships that could be construed as a potential conflict of interest.

## Footnotes

1 http://www.genome.jp/kegg/

2 http://www.nist.gov/index.html

3 http://www.metaboanalyst.ca

## Supplementary Material

TABLE S1 The relative abundance of bacterial taxa significantly differentiated between Con and EO group. Con, the basal diet; EO, the basal diet supplemented with essential oils.
